# Three‐Dimensional Characterization of the Collagen–Hydroxyapatite Interaction During Heterotopic Ossification in Healing Rat Achilles Tendons

**DOI:** 10.1002/smsc.202500644

**Published:** 2026-06-20

**Authors:** Kunal Sharma, Isabella Silva Barreto, Hector Dejea, Irene Rodriguez Fernandez, Dario Ferreira Sanchez, Pernilla Eliasson, Maria Pierantoni, Hanna Isaksson

**Affiliations:** ^1^ Department of Biomedical Engineering Lund University Lund Sweden; ^2^ MAX IV Laboratory Lund University Lund Sweden; ^3^ Center for Photon Science Paul Scherrer Institute Villigen Switzerland; ^4^ Institute for Biomedical Engineering ETH Zürich Zürich Switzerland; ^5^ Department of Biomedical and Clinical Sciences Linköping University Linköping Sweden; ^6^ Department of Orthopaedics Sahlgrenska University Hospital Gothenburg Sweden; ^7^ Department of Orthopaedics Sahlgrenska Academy Gothenburg University Gothenburg Sweden

**Keywords:** biomineralization, material science, nanoscale, nanostructures, X‐ray scattering

## Abstract

Pathological ossification is seen affecting different organs across the body. This includes the Achilles tendon, where the healing process after rupture can be impaired by heterotopic ossifications (HO). The formation pathways for HO in tendons are still debated, and one reason for this is our limited knowledge of their structure and composition in 3D. This study utilizes a multimodal 3D characterization approach to study HO and the surrounding healing tissue at 3‐, 6‐, and 12‐week post‐transection in rat Achilles tendons. Microcomputed tomography provided 3D microstructural information. Small‐ and wide‐angle scattering tensor tomography contributed with combined collagen and hydroxyapatite (HA) 3D nanostructural information. Combined 3D X‐ray diffraction and fluorescence provided high‐resolution structural dimensions of HA crystallites, and chemical composition of both the deposits and the surrounding healing tendon tissue. Morphologically, dense mineralized tissue appeared more fibrous, with higher co‐localized quantities of Zn present internally, while porous regions of HO deposits had Zn localization primarily at the boundary. Collagen d‐spacing was higher within HO deposits than in the surrounding collagenous tendon tissue. Collagen misalignment close to the HO boundary supports the notion of tissue mineralization occurring prior to the full assembly of the healing tendon network. This methodology can be extrapolated to investigate pathological ossification in other tissues at the nanoscale while capturing the heterogeneity of entire specimens.

## Graphical abstract

Multimodal 3D characterization of heterotopic ossification (HO) during tendon healing showed different morphologies at the microscale through microcomputed tomography. Small‐ and wide‐angle scattering tensor tomography contributed with 3D nanostructural information of both collagen and hydroxyapatite (HA), demonstrating a disorganized healing matrix and HO formation. Combined 3D X‐ray diffraction and fluorescence provided high‐resolution structural dimensions of HA crystallites, and chemical composition of the deposits, identifying key trace elements such as Zinc and Iron.

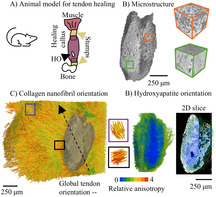

## 1 Introduction

Connective tissues such as tendons and ligaments are commonly injured [1, 2], and healing after a tendon rupture is a slow process. After Achilles tendon rupture, a mesh‐like collagen network forms, which in time rearranges into a more uniformly oriented collagen structure. However, in most cases, the tendon does not fully regain its mechanical properties. One complication associated with poor healing outcomes are pathological ossifications in the tendon which affect 14%–28% of patients [3, 4, 5, 6]. This could be linked to reduced motion and increased pain after healing caused by heterotopic ossification (HO) [5]. HO deposits appear as dense “bone‐like” objects; however, there is limited knowledge about their origin, structure, and composition, and there are currently no preventative measures. HO has been shown to occur during tendon healing or in tendinopathy, and is possibly linked to inflammation [7, 8, 9, 10]. Bone mineralization occurs in many situations in the body, including embryonic long bone growth [11, 12], bone fracture repair [13], and HO [14]. In all cases, these involve a collection of highly regulated events that depend on the specific environment. Hydroxyapatite (HA) crystals are deposited within the gap zones of a collagen template and across fibrils, creating a well‐aligned composite material where the HA provides stiffness and strength, and collagen provides flexibility and toughness [15, 16, 17]. In HO, mineral deposition occurs through endochondral ossification [7], similar to embryonic long bone development and repair processes.

Animal models are commonly used to investigate the formation of HO deposits during tendon healing under controlled conditions [8, 9, 18, 19, 20]. Our previous studies on HO in transected healing rat Achilles tendons demonstrated a time‐dependent HO deposit formation, originating from preinjury deposits, which act as templates for further mineralization. The HO extends to the healing tissue, occupying up to 10% of the tendon volume after 20 weeks [21]. Interestingly, it was observed that pre‐ and post‐injury deposits are characterized by different microstructures that make the two clearly distinguishable [21]. We also found that mineralization occurred in areas with high proteoglycan content and was associated with the presence of round chondrocyte‐like cells [21]. These studies observed the spontaneous formation of HO in both healing and intact tendons [22]. Thus, it was not induced, but rather an observation that led us to further analyze the 2D nanoscale properties of HO deposits [14]. Further, recent studies by others investigating the mineralization of tendons and ligaments in vertebrates identified mineral nucleation both between and across collagen fibrils [21, 23, 24]. Therefore, investigating the tissue's composition and structural arrangement at the micro and nanoscale is essential to fill the current gap in knowledge that exists around HO deposition in healing tendons.

During tendon healing, an initial disorganized matrix of collagen type III is deposited, which is later replaced by more aligned collagen type I [25, 26]. Trace elements of Iron (Fe) have been linked with procollagen enzymes required for precursors in collagen formation. Histological data suggest that during these phases of healing, HO occurs in Achilles tendons through endochondral ossification [7], which includes the formation of a temporary cartilaginous template that is later mineralized. The presence of Zn at the boundaries of mineralizing bone tissues has previously been reported during growth, embryonic development, and fracture healing [11, 13, 27, 28, 29, 30, 31]. Our recent study on HO deposits in rat tendons demonstrated the presence of Zinc (Zn) and Iron (Fe) as trace elements at the mineralization front preceding crystallized HA [14]. However, while our previous study focused on the mineralized HO and investigated its structure across length scales, it was primarily performed in 2D. In that study, we found a necessity for 3D investigation due to the heterogeneous nature of the tissue [14]. Furthermore, a recent review of hierarchical materials confirmed the need for hierarchical investigations of biological materials to provide information regarding their structure and function in 3D [32]. Thus, little is known about the 3D collagen arrangement within and surrounding HO deposits in Achilles tendons and the spatial transition between collagenous matrix and mineralized tissue. To that extent, 3D characterization techniques are necessary to elucidate the structure and composition of both collagen and HA, and their interactions at the nanoscale.

Microcomputed tomography (microCT) allows for the visualization of the 3D meso‐ and microarchitecture of the complex mineralized deposits. To probe the nanometer scale, powerful synchrotron light sources allow for both structure and composition to be investigated in tendon tissues [33]. Small‐ and wide‐angle scattering tensor tomography (SASTT/WASTT) was recently developed and yields nanoscale information for each voxel in millimeter‐sized samples, allowing for the study of the tissue in 3D without the need for sectioning [34, 35, 36]. SASTT/WASTT data have been performed on other mineralized tissues [37, 38, 39, 40, 41], and for collagenous tissues [42] separately, but not for pathological ossification or the combined properties of mineralized tissue and surrounding collagen matrix. Similarly, X‐ray diffraction (XRD) and X‐ray fluorescence (XRF) have long been state‐of‐the‐art in characterizing mineralized tissues in 2D. However, only recently, 3D‐XRD and 3D‐XRF were used to study nanoscale structure and composition in mineralized tissues [43, 44]. Limitations still exist with the self‐absorption of lower atomic number elements. However, the unprecedented 3D investigation yields information regarding the heterogeneity of the tissue and provides a more comprehensive view of the sample properties.

In this work, we apply a multimodal approach that allows the study of the structure and composition of the tissue in millimeter‐sized samples, providing a more comprehensive representation of the tissue properties. This study aims to explore the structure, elemental composition, and orientation at the nano and microscale in 3D of entire HO deposits and surrounding collagen tissue in healing rat Achilles tendons, to link HA deposition to the healing outcomes of the collagen network.

## 2 Methods

### 2.1 Animal Model

This study uses samples from a previous study that investigated the progression of HO through phase‐contrast synchrotron imaging in 29 rats (female Sprague–Dawley rats, 12‐weeks old, Janvier, Le Genest‐Saint‐Isle, France) where the Achilles tendon was harvested at different time points from 1‐week to 20‐weeks after Achilles tendon transection [21]. In all rats, the tendon was surgically transected and allowed to heal spontaneously [21, 26]. In a different study, a subset of the samples (*n* = 14) was fixed in formalin for 48 h and embedded in Ploy/Bed 812 (DMP‐30 Kit, Polysciences Europe GmbH), in order to quantify the structure and elemental composition using a range of 2D characterization techniques at the micro‐ and nanoscale to elucidate the potential pathways of HO formation [14]. In the current study, Achilles tendons from 3 of those rats, representing 3‐, 6‐, and 12‐week post‐injury, were used. Additionally, a calcaneal bone sample was obtained from the 12‐week sample as a reference. The experiment adhered to institutional guidelines for care and treatment of laboratory animals and was approved by the Regional Ethics Committee for animal experiments in Linköping, Sweden (Jordbruksverket, ID1424).

### 2.2 MicroCT

Embedded samples were trimmed down with a low‐speed saw (Buehler, ISOMET) to approximately 1 × 1 × 10 mm^3^, isolating the HO deposits. Samples were then imaged with X‐ray tomography (Zeiss XRM 520, Germany) at 80 kV, 7W, and with a Le1 filter. The number of projections was 1601, with a 4x detector and a binning factor of 2. The sample‐to‐detector distance was 36.79 mm, and the sample‐to‐source distance was 10.53 mm, resulting in a final isotropic voxel size of 1.5 μm.

### 2.3 SASTT/WASTT

Simultaneous SASTT and WASTT measurements were carried out at the cSAXS beamline of the Swiss Light Source at the Paul Scherrer Institute (Switzerland) with a monochromatic beam of 12.4 keV, with a 9 × 25.5 μm^2^ beam size. The SAXS detector (Pilatus 2 M, Dectris, Switzerland) was placed at the end of a flight tube at a distance of 7.1238 m from the sample to record a *q*‐range of 0.01–1.8 nm^−1^. The direct beam intensity is measured with a beamstop in the flight tube. The WAXS detector (300k‐W Pilatus, Dectris, Switzerland) was placed at a sample‐to‐detector distance of 639.5 mm at an angle of 15.3° with respect to the beam path to access a *q*‐range of 1.55–29 nm^−1^. The samples were mounted on a needle and placed onto a goniometer. The samples were visually aligned and centered using an optical microscope (IDS camera, field of view 1.8 mm).

The samples were mapped in x and y (fly scan in y‐direction) with a step size of 25 μm and an exposure time of 50 ms. Projections were captured at 7 (3‐week sample), 6 (6‐, 12‐week sample), or 5 (bone sample) equally spaced tilt angles (α) between 0° and 45° in the direction of the beam, and at rotation angles (θ) around the *y*‐axis. At *α* = 0°, *θ* was equally spaced between 0° and 180°, for *α* ≠ 0° *θ* was equally spaced between 0° and 360°. The number of tilt angles was modified to accommodate the imaging of all samples within the time constraints of the allocated beamtime.

All data were integrated radially using the cSAXS Matlab package [45]. See Figure S1 for an example of radial integration 1D *I(q)* curves for small‐ and wide‐ angle X‐ray scattering patterns. Details of the SASTT reconstruction procedure can be found elsewhere [34, 37, 46]. Briefly, to evaluate fibril and HA orientation, the reciprocal space maps in each voxel are described as a series of spherical harmonics of orders *m* = [0,0,0,0], and degrees *l* = [0,2,4,6], and reconstructed with the recently developed spherical integral geometric tensor tomography (SIGTT) and software package MUMOTT v0.2 [34]. See Table S2 for more details regarding acquisition parameters for each sample. Orientational analysis was performed for two q‐regions: collagen fibril orientation (*q* = 0.08–0.1 nm^−1^) and mineral platelet orientation (*q* = 0.5–0.6 nm^−1^). Further, the anisotropy is measured as the degree of orientation, which is analyzed as the ratio between the anisotropic scattering and the total scattering. The value of anisotropy therefore, provides an indication of the fibril alignment within each voxel.

To reconstruct the 1D scattering curves in each voxel, the zonal spherical harmonics tensor tomography method was utilized with the band limit set to 0, yielding an isotropic spherical function removing orientational dependence [37, 39]. Fibril and HA structural parameters were reconstructed per voxel [47]. 1^st^ collagen peak scattering was analyzed with 40 logarithmically spaced *q*‐bins for the *q*‐range *q* = 0.08–0.1 nm^−1^, mineral platelet thickness (*T*‐parameter) was analyzed with 200 logarithmically spaced *q*‐bins for the *q*‐range *q* = 0.3–1.7 nm^−1^. Finally, to quantify the Scherrer width for the (002) and (310) reflections 50 logarithmically spaced *q*‐bins were used in the *q*‐range *q* = 17–19.5 nm^−1^, and *q* = 25–28.8 nm^−1^ respectively. The reconstructed 1D curves were then fit with Gaussians for further analysis with in‐house Matlab scripts [48]. The Gaussian fit of the collagen peak was used to obtain peak position (fibrillar *d*‐spacing), peak area (relative fibril quantity), and full‐width at half maximum (FWHM). The mineral shoulder (*q* = 0.3–1.7 nm^−1^) was fitted through an iterative nonlinear least squares method to provide the HA platelet thickness (*T*‐parameter), following the method described elsewhere [49, 50]. Finally, the size of the crystallites (the cumulative arrangement of crystals), *L*‐parameter (002 reflection, size along *c*‐axis), and *W*‐parameter (310 reflection, size along the *ab*‐plane) were calculated based on the Scherrer equation [51, 52]. The *L*‐ and *W*‐parameter sizes were combined into an aspect ratio (*W/L*) to yield information regarding the shape of the crystallites in all samples.

### 2.4 3D‐XRD/3D‐XRF

The embedded samples were further trimmed down to approximately 250 × 250 μm^2^ using a Nano lathe (NanoMAX beamline, MAX IV; settings rpm: 1200, 0.3 mm carbide endmill). The region of interest (ROI) were selected where active mineralization was anticipated from the acquired microCT images. Simultaneous 3D‐XRD and XRF measurements were carried out at the microXAS beamline, Paul Scherrer Institute (PSI, Switzerland) with an X‐ray energy of 18.1 keV, 2 μm^2^ beam size, 2 μm step size, and 100 ms exposure time. The XRD detector (Eiger 4 M, Dectris, Switzerland) was placed at a sample‐to‐detector distance of 140 mm, resulting in a *q*‐range of 0.025–63.22 nm^−1^. Four XRF detectors (FalconX, XIA LLC, USA) were placed 20 mm from the sample 90°, apart from each other. Each sample was scanned at equally spaced rotation steps of 1.35° (between 0° and 360°), resulting in tomograms with 133 projections and 3 μm on‐plane pixel size. To cover the sample size vertically, a tomogram was acquired every 3 μm, thus resulting in a final isotropic voxel size of 3 μm. See Table S3 for more details regarding acquisition parameters.

All acquired diffraction patterns were radially integrated and corrected by the transmission signal recorded with a diode in the beam stop to remove beam fluctuations. The hot and cold pixels were masked away by replacing the pixel value with neighboring pixels to avoid streak artifacts in the reconstruction. Both diffraction and XRF sinograms were reconstructed, and using a simultaneous inverse Radon transform algorithm, a reverse analysis was carried out to obtain the crystalline phase and chemical composition [53, 54].

The reconstructed XRD tomograms were further analyzed with in‐house MATLAB packages (R2021a, MathWorks Inc., USA) [48] for defined q‐ranges for the (002) and (310) reflections to provide structural information regarding the HA dimensions. The peaks of interest were fit with Gaussian curves, and the crystallite dimensions were obtained using the Scherrer equation [51, 52]. The mineral area was computed by multiplying the size of crystallites along the *c*‐axis and along the *ab*‐plane. The reconstructed XRF volumes were analyzed for the elements of interest, i.e., Calcium (Ca), Zinc (Zn), Iron (Fe), and Strontium (Sr), using PyMCA [55] following our previous approach [13, 14]. Self‐absorption is a common challenge during XRF, where part of the fluorescence signal from the sample is absorbed by the sample itself and cannot therefore be captured by the detectors. This is crucial for low‐energy fluorescence, such as Ca (*K*‐emission: 3.69 and 4.01 keV). The 3D reconstructions pointed to a strong self‐absorption gradient of Ca from the border to the center of the sample (Figure S4). For this reason, Ca was not quantified. Instead, Strontium (Sr) was used as it is known to be a trace element in bone and is correlated to Ca with an energy of 16.12 keV [27, 49]. Sr substitutes approximately 3.5% of Ca in the human body and can thus be used as a proxy for Ca localization [56], both elements are incorporated into bone at a similar rate [57]. The XRF maps were used to create 3D renderings to better visualize the presence of elements and correlated to the HA scattering to provide insights into the role of Sr, Zn, and Fe in HO.

### 2.5 Radiation Damage

Radiation damage is known to affect biological tissues. Structural changes in the collagen fibrils have been reported at around 70 kGy [42, 58]. The radiation dose was assessed for the acquired tomographies, the SASTT/WASTT experiment, and the consecutive 3D‐XRD/XRF experiment. To estimate the dose on the sample, the following equation was used

(1) $$d=\frac{EI_{0}\tau A}{\text{Δ}x\text{Δ}y\text{Δ}z\cdot \rho }$$

where *d* is the dose, *E* is the energy in joules, *I*_0_ is the incident flux in photons/s, *τ* is the exposure time in seconds, and *A* is the absorption ratio. ΔxΔyΔz is the volume illuminated by the beam, and *ρ* is the mass density of the sample (for collagen, which is the more sensitive tissue, it is 1120 kg/m^3^) [42]. For the tomographies, the resulting total dose on the sample was found to be minor. For the SASTT/WASTT experiment, the resulting total radiation dose was in the range of 4.16–7.48 kGy. The consecutive 3D‐XRD/XRF experiment resulted in an additional dose in the range of 1.38–2.27 kGy. Thus, across all experimental sessions, the accumulated dose on each sample was below 10 kGy.

### 2.6 Data Analysis

The mean scattering intensity of the SASTT acquisition was used to threshold and separate the mineralized tissue (>4000 A.U.) from the surrounding collagen. The two masks were created for each sample and used to quantitatively compare the structural properties (*d*‐spacing, FWHM, crystallite dimensions) of mineralized tissues across samples, and against surrounding collagen tissue. 2D slices from tomography volumes were used to overlay the SASTT orientation results to extrapolate a relationship between collagen and HA orientations and morphological features. The same 2D slices were used to elaborate on the localization of higher *T*‐parameter values with present morphological features. 3D renderings of the crystallite structural parameters from 3D‐XRD were visualized and spatially correlated to the analyzed elemental information from 3D‐XRF. Visualizing the combined 3D rendering of Sr, Zn, and Fe allows for a spatial understanding of the elemental presence in HO deposits at the investigated healing time points. Simultaneously, visualizing the combined structural (*L*‐ x *W*‐parameter) crystallite dimensions allowed for a comparative identification of the present elements within the same spatial area as larger crystallites.

## 3 Results

### 3.1 HO Deposits are Characterized by Two Possible Micromorphologies and Differ From Bone

**FIGURE 1:**
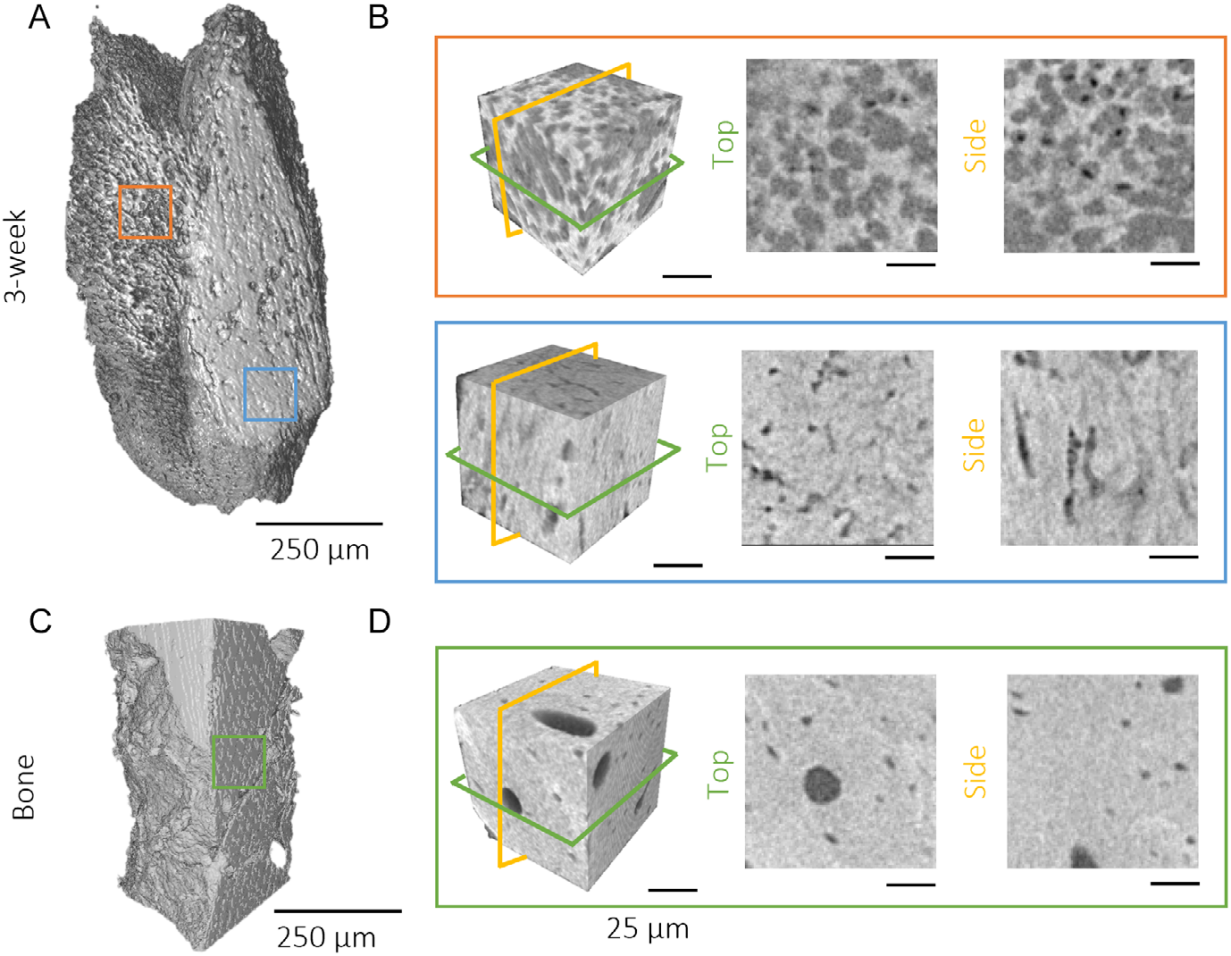
MicroCT images showing HO deposits 3‐weeks after transection, and the bone reference from the calcaneal bone. 3D renderings of mineralized tissue of (A) HO‐deposit after 3‐weeks, and (C) bone reference. Sub‐volumes of interest (B) within the HO deposit with cross and longitudinal slices showing porous regions (orange), and fiber‐like internal structure (blue), and (D) bone (green).

The microCT volumes showed two distinct morphologies present within the HO deposit at 3‐weeks (Figure 1A). One sub‐volume was extremely porous with small voids of approximately 10–15 μm in diameter (Figure 1B, orange), while the other sub‐volume had a characteristic fibrous pattern possibly indicating that ossification occurred along the tendon collagen fibers (Figure 1B, blue). A similar porous structure was observed in the 6‐week sample, while the 12‐week deposit was mostly characterized by a fibrous structure (Figure S5). The internal morphology of all HO deposits was more heterogeneous compared to the bone reference sample, in which the blood vessels and lacunae were visible inside the compact tissue (Figure 1D).

### 3.2 Collagen Orientation is Disorganized Close to HO Deposit Boundary

**FIGURE 2:**
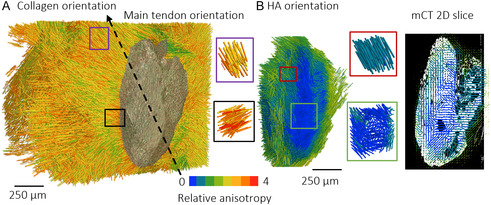
Collagen and HA quantification through SAXS. Main collagen orientation was estimated from previous tomography experiments of these samples [21]. Glyph render of 3‐week sample showing main orientation (glyph direction), mean scattering size (glyph size) relating to amount of material present, and relative anisotropy (color scale) exemplifying the degree of alignment for (A) collagen with 3D rendering of the microCT data to show HO deposit location, and (B) HA which is also overlayed on a corresponding 2D slice from tomographic imaging. Boxes show zoom‐ins for collagen scattering from the surrounding collagen tissue (purple), and close to the HO deposit boundary (black), and from HA scattering the porous region (red), and dense internal structure (green).

SASTT analysis of the 3‐week sample showed regions with less collagen content around the immediate boundary of the HO deposits (Figure 2A, thickness of glyphs in black box), compared to regions further away (Figure 2A, purple box). Similarly, less scattering from HA was present at the HO boundary, suggesting a lower presence of crystalline structures or a less mature ossification state (Figure 2B). Furthermore, the relative anisotropy (visualized as a change in color) was higher in the collagen located at the boundary. Therefore, the boundary region between the HO deposit and surrounding tendon tissue has a larger variation in orientation (Figure 2A, change in color in the black box) from the main collagen axis (seen as the direction of the glyphs) when compared to the bulk of the tendon (Figure 2A, purple box). In the denser fiber‐like regions of the HO deposit, the HA crystallites are orientated in a twisting pattern (Figure 2B, green box). However, in the porous region, the crystallites show a more aligned orientation (Figure 2B, glyph orientation). These findings were consistent with what was observed for the other HO deposits ( Figure S6), and their respective main tendon orientations are visualized in Figure S7.

### 3.3 Mineralized Collagen Inside HO Deposits Results in a Greater *d*‐Spacing

**FIGURE 3:**
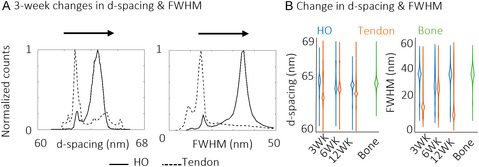
Collagen structural parameter quantification. (A) Fibril *d*‐spacing, and heterogeneity (FWHM) for the mineralized region (solid line), and surrounding healing tendon collagen (dashed line) in the 3‐week sample. (B) Change in *d*‐spacing (left) and FWHM (right) for all samples with violin‐plots showing the distribution of values for HO (blue), tendon (orange), and reference values for bone (green).

The *d*‐spacing, relating to the structural arrangement of collagen molecules, was ∼3% higher (between 0.9 and 1.6 nm difference) for the HO deposits (3‐ and 12‐week) compared to the tendon bulk collagenous tissue (Figure 3A, left). The FWHM of the collagen peaks was ∼20 nm greater inside the HO deposit compared to the bulk collagenous tendon tissue, suggesting higher heterogeneity in collagen arrangement (Figure 3A, right). While the 6‐week sample did not show any increase in *d*‐spacing or FWHM (Figure 3B). The average *d*‐spacing (64.1 ± 0.4 nm) of HO deposits was consistent with bone reference values (64.1 nm). While the FWHM (heterogeneity, 35.3 ± 1.5 nm) for all HO deposits was not largely different from that of the bone sample (37.5 nm).

### 3.4 Structural Parameters of HA Crystallites are not Uniform Within the HO Deposits

**FIGURE 4:**
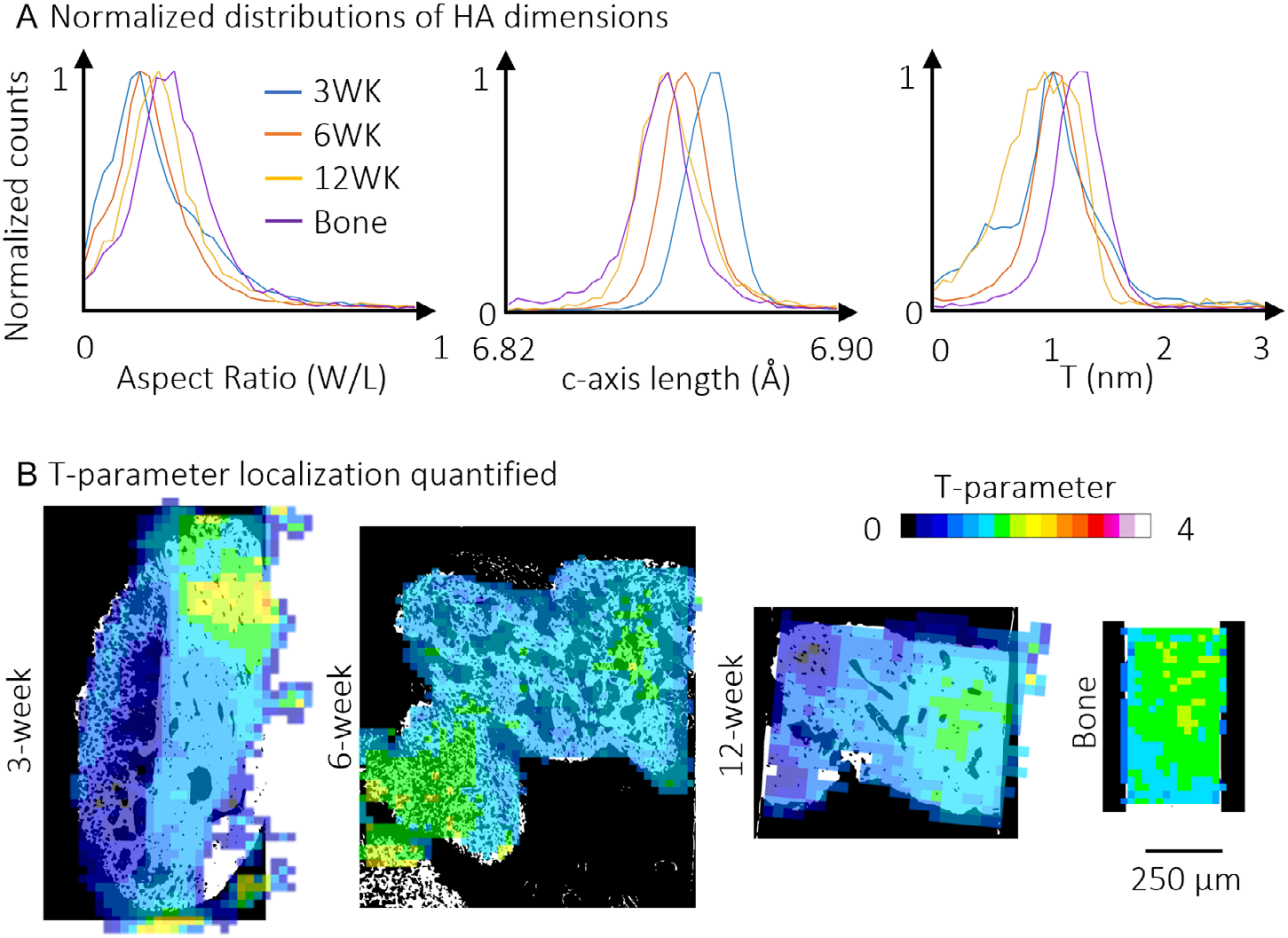
Crystallographic analysis of HA scattering through SAXS (mineral particle thickness), and WAXS. (A) Normalized distributions of HA dimensions for: ratio of *W/L* relating to crystallite aspect ratio (left), HA crystal size along the *c*‐axis (middle), and *T*‐parameter relating to thickness of crystallites (right). (B) *T*‐parameter maps overlayed on 2D slices from tomographic images.

Through WASTT and the Scherrer equation, the ratio of the *W*‐parameter (size along *ab*‐plane), and *L*‐parameter (size along *c*‐axis) was on average 5%–7% lower for the HO deposits when compared to bone, suggesting a more needle‐like shape of crystallites (Figure 4A, left). An opposite trend was observed in the *c*‐axis lattice size of individual HA crystals on average, where the 3‐week HO deposit exhibited the largest size along the *c*‐axis, 6.87 Å, while bone was lowest, 6.85 Å (Figure 4A, middle). Finally, the *T*‐parameter, related to the thickness of HA crystallites, was on average 15% lower in all HO deposits when compared to the bone reference sample (Figure 4A, right). Furthermore, the *T*‐parameter data showed regions with higher values (stagger of multiple crystals), indicating different levels of maturation within the HO deposits (Figure 4B). Alignment with the microCT images showed that higher *T*‐parameter values are localized within denser (fibrous) regions of the 3‐ and 12‐week samples. The 6‐week sample, which was characterized by a porous structure, contained more than one area of higher *T*‐parameter values. Furthermore, across all samples, lower *T*‐parameter values were observed at the boundary of the HO deposits, while in bone, the *T*‐parameter was higher and more homogenously distributed.

### 3.5 3D Elemental Distribution in HO Deposits Correlate With Nano‐ and Microstructural Features

**FIGURE 5:**
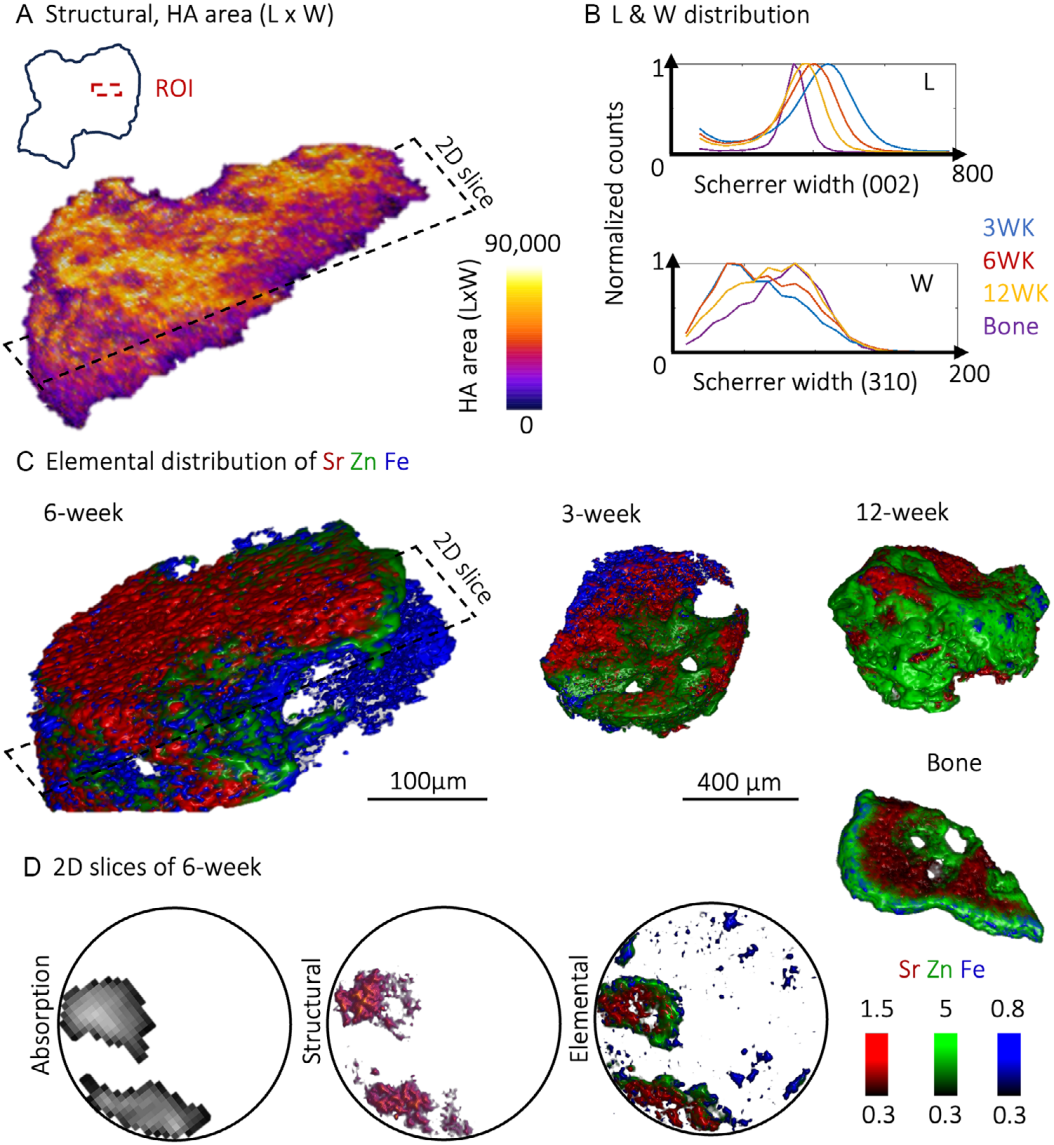
Structural and elemental distributions acquired by 3D‐XRD/XRF. (A) Structural HA area based on *L x W*, with a schematic showing the ROI within the 6‐week sample. (B) Structural distributions based on Scherrer width of the (002) reflection (*L*, apparent crystallite size along *c*‐axis, top), and (310) reflection (*W*, apparent crystallite size along the *ab*‐plane, bottom) for all samples and bone reference. (C) Combined elemental distributions of Sr (red), Zn (green), and Fe (blue) for all samples and the calcaneal bone reference. (D) 2D slices of the absorption (tomography), structural (XRD), and elemental (XRF) results for the 6‐week sample showing the presence of Fe in the tissue further from the already mineralized area.

The higher resolution 3D‐XRD/XRF results for a ROI enabled correlation between the structural HA crystallite dimensions and the elemental presence. Lower HA crystallite area (*L x W*) was found closer to the HO deposit boundary across all samples (Figure 5A and Figure S8). The size distribution of crystallites along the *c*‐axis was higher in HO deposits compared to bone, as indicated by the higher on average *L*‐parameter, whereas the opposite was observed in the scattering along the *ab*‐plane, where HO deposits had on average a smaller *W*‐parameter compared to bone (Figure 5B). These higher resolution results are in line with what was observed for the entire deposit from tensor tomography, suggesting a more needle‐like shape of crystallites in HO deposits. Zn was localized to the boundary of HO deposits and surrounding larger voids in all samples (Figure 5C). While the 12‐week sample and bone contained a low amount of Fe across the entire sample, which was diffusely distributed. Interestingly, in the 6‐week sample, Fe was localized at the HO deposit boundary and in the neighboring healing tendon callus (collagenous matrix), where no HA was detected (Figure 5D).

## 4 Discussion

This study investigated the 3D structure and composition of HO deposits in relation to the surrounding collagenous matrix. Structural differences such as higher *d*‐spacing and heterogeneity where observed when comparing the collagen network inside HO deposits to the surrounding healing tendon matrix. This study furthered our understanding of how Zn and Fe participate in the mineralization process by showing their presence localized at the boundaries of HO deposits, with Fe present outside the mineralized regions. Studies in 2D have reported Zn localization at the boundary [11, 12, 13, 14], and the current study adds 3D information by showing orientational and compositional heterogeneity of the HO deposits, which also affect the collagen network structure. Capturing the 3D structure and composition of the tissue allows for a better understanding of the relation between collagen regeneration during tendon healing and HO.

### 4.1 Collagen Nanostructure Within HO deposits is Less Organized Than in the Surrounding Soft Tendon Tissue

Tomography results revealed HO deposits with two distinct morphologies: one appearing more fiber‐like, and the other, more prevalent, porous with voids of <15 μm in diameter. We have previously reported the fiber‐like morphology in naturally occurring HO deposits in rat Achilles tendons, where the deposits were characterized by an ellipsoidal shape [22]. The porous morphology, on the other hand, was found to be present in ossification of healing tendons, initially growing on the fibrous preinjury HO deposits in the intact stump, then forming in regenerating tissue in the callus, and, after 20 weeks of healing, merging into large structures occupying up to 10% of the tendon volume [21]. First of all, similarly to what we found before, fiber‐like morphology was observed in the 3‐week sample, which was also characterized by a porous structure on the periphery of the deposit. In line with our previous findings, this could indicate an active mineralization site on the pre‐existing HO deposit present in the Achilles tendon from before transection. Second, the porous nature of most of the HO deposits investigated in this study suggests that they were primarily formed during the healing process, and not before tendon transection. In line with these observations, at the nanoscale, samples from different time points of healing revealed different collagen structural arrangements within the HO deposit and the surrounding healing tendon matrix. Fibrillar orientation was more heterogeneous (greater FWHM) at the HO deposit boundary, combined with a different orientation to the main collagen axis observed further away in the tendon matrix (Figure 3B). This observation can be explained considering that, during tendon healing, a callus is formed out of initially collagen type III, which is less organized than the later remodeled collagen type I [25, 26]. Consequently, collagen orientational differences could be due to collagen retaining the less organized structure of collagen type III present at early tissue healing stages, and further reorientation was possibly hindered by the deposited hard mineral, whereas the surrounding nonmineralized tissue can continue to heal, remodel into collagen type I, and reorient. Furthermore, the large difference in orientation at the boundary of the HO deposit and the surrounding tendon matrix could be due to early formation of HO deposits originating in the intact stumps, where the tendon tissue is more aligned [21]. In agreement with the hypothesized HO formation hindering collagen reorientation, a previous study, where HO deposits were studied at the nanoscale through focused ion beam scanning electron microscopy (FIB‐SEM), observed tessellation and the tesselles were believed to form around mineral foci disrupting the collagen network [23, 59, 60]. This tessellation was shown to act across fibrils, which would affect the orientation of collagen [23]. Therefore, the exact mechanisms behind the difference in collagen orientation are complicated to differentiate. On the one hand, normal animal movement during healing may promote collagen remodeling while HO deposits are already present within the matrix. On the other hand, the mineralization process itself may physically constrain the tissue and effectively “lock in” a less organized collagen architecture if mineral deposition occurs before collagen reorganization is complete. Collagen fibril changes can also be quantified through the change in packing of collagen molecules (*d*‐spacing). This study found the *d*‐spacing within HO deposits to be ∼3% greater than in the surrounding tendon tissue, and more similar to the values of the investigated bone sample. The *d*‐spacing of the tissue can be affected by a number of different factors, including mineralization [17], hydration of the tissue [48], and others. A likely reason for the difference in *d*‐spacing could be that HA is arranged between the gaps in collagen molecules [61, 62], with the tissue becoming mineralized, the collagen *d*‐spacing is increased within the HO deposits compared to the surrounding nonmineralized tendon tissue. A similar trend has been observed in mineralized collagen tissues such as turkey tendons where the *d*‐spacing of collagen molecules was reported to increase 1%–2% in the mineralized regions, compared to the nonmineralized tissue [17]. However, another model based on unmineralized turkey tendons hydrated the samples in SrCO_3_ solution to induce deposition and monitor the process. The study proposed a decrease in *d*‐spacing, which was attributed to contractile forces generated by the newly occurring mineralization [15]. One explanation for the results in this study could be that the onset of mineralization could be occurring both in the gap regions and across fibrils, which would constrict their ability to reorient as expected during tissue healing. Moreover, the *d*‐spacing is most likely also affected by the hydration state, with water molecules being either pushed out or absorbed during nucleation, growth, and development [63, 64]. A lower hydration would therefore result in a decrease in *d*‐spacing. However, whether dehydration is induced or is the result of mineralization is hard to differentiate. Moreover, the proposed models for collagen structural changes during mineralization are based on intact tissues, whereas this study is conducted on healing tendons, where the formation process and structure of collagen are clearly different from intact tendons [26, 42, 65].

### 4.2 HO Deposits are Characterized by Needle‐Like Crystallites

Mineralization is a complex process. One parameter that is used as a proxy for the maturation state of bone is the *T*‐parameter, which is related to the thickness of mineral particles. In this study, the *T*‐parameter ranged on average between 1 and 1.3 nm in the HO deposits, while it was 1.5 nm in the reference bone (Figure 4B). Previous studies on adult and cortical bone found *T*‐parameter values reaching 2.5 nm, which is higher than what was observed in this study [11, 13, 50, 51, 66, 67]. Interestingly, studies on embryonic bone observed lower values around 1.6 nm, which is well in line with the results from this study [12]. Higher *T*‐parameter values were localized within one region for 3‐ and 12‐week samples, and in multiple regions for the 6‐week sample, whereas bone contained larger values overall, more homogeneously (Figure 4B). The 6‐week sample containing multiple regions of high *T*‐parameter values (>1 nm) could be the result of 2 smaller HO deposits merging into one larger structure, thereby containing two original nucleation sites. This is in line with observations from our previous study, where the merging of HO deposits at later healing time points was described [21]. Furthermore, in this study, the ratio of *L*‐ and *W*‐parameters, which relate to the size of crystallites along the *c*‐axis and across the *ab*‐plane, respectively, showed the crystallites within the HO deposits to be more needle‐shaped than for the reference bone, which exhibited more oval‐like dimensions. Literature shows that preferential mineralization in forming bones occurs along collagen fibrils, eventually leading to cross‐fibrillar mineralization in animals and humans [23, 24, 67, 68]. The 12‐week HO deposit contained, on average, broader crystallites (greater size along the *ab*‐plane compared to 3‐ and 6‐week samples) and appeared less needle‐like, thus being more similar to the bone reference and possibly indicating an older HO deposit. One explanation for this could be that after a certain stage of healing, the extrafibrillar mineralization takes over [69], or it could be the result of transverse stacking of mineral particles across several fibrils [39].

### 4.3 Traces of Zn and Fe Indicate Active Mineralization

We and others have previously reported traces of Zn and Fe to be related to the mineralization process in bones and HO deposits [12, 13, 14]. Zn co‐localization at the boundary has been found in mineralized tissues for rats, humans, and during embryonic bone development [11, 13, 14, 27, 28, 29, 30, 70, 71, 72, 73]. Zn is a known stimulator for osteoblasts (bone‐forming cells), while simultaneously inhibiting osteoclasts (bone‐resorbing cells). In this study, Zn was present at the boundary and within the HO deposits, suggesting a similar role in HO. Zn has previously been shown to accumulate in cement lines, where it was believed to attach directly to HA or proteins [27]. In this study, Zn was seen around larger voids inside the HO deposits, and its presence internally could be a sign of further mineralization. Another study with this animal model found large voids in HO deposits to close over time and become mineralized [21]. However, the 6‐week sample contained Zn primarily on the boundary of the HO deposit (Figure 5C), with none present internally, which would more strongly support the notion of Zn partaking in growth that occurs outwards first concentrating on merging HO deposits initially at early time points [21, 22].

Fe was largely seen in the 3‐ and 6‐week HO deposits, coinciding with regions with low to no mineral content. One explanation for Fe being located outside of mineralized regions is its potential role in the breakdown of collagen [74], or vascularization [75, 76] of the cartilage template that is laid down prior to chondrocyte hypertrophy and later mineralization [76, 77]. Mineralization is a complex process therefore, it is hard to differentiate the roles of trace elements as they could be attributed to mineral formation and collagen breakdown, and to any stage within these 2 processes. Thus, there is still a need for further testing to target and capture the nature of the mineralization process, especially HO in both intact and healing tissues.

### 4.4 Limitations

It is important to note some limitations of this study. First, due to competitive and limited synchrotron experimental times, only one sample per healing time point was analyzed with the complex 3D techniques. The current advancements in 4^th^ generation synchrotron sources will allow for faster measurements, which in turn will enable more samples to be measured in the allocated time. The second limitation is that the exact age of the HO deposits is unknown. As new deposits are formed at all times during healing, the age of each HO deposit unfortunately does not necessarily correlate with the healing time point. The HO deposits could have formed on preinjury deposits (that function as mineralization seeds) and some not, which makes it difficult to elucidate chronological patterns in mineral formation and maturation. To that extent, a more controlled model of mineralization is necessary to further elucidate the exact time points for nucleation, mineral deposition, and maturation.

## 5 Conclusion

The three‐dimensional structure and composition of HO deposits and their interaction with the surrounding collagenous matrix demonstrated structural differences (higher *d*‐spacing and heterogeneity) in the collagen network inside HO deposits when compared to the surrounding healing matrix. Furthermore, Zn and Fe (known trace elements in the mineralization process) were found at the boundaries of HO deposits, indicating that mineralization is ongoing and follows known bone processes. These findings lay the fundamental grounds to understanding the formation of HO deposits, and hopefully provide a stepping stone for further investigations into preventative measures.

## Author Contributions

**Kunal Sharma**: data curation (lead), formal analysis (lead), investigation (equal), methodology (equal), visualization (lead), writing – original draft (lead). **Isabella Silva Barreto**: conceptualization (equal), data curation (equal), formal analysis (supporting), investigation (equal), methodology (equal), validation (equal), writing review & editing (equal). **Hector Dejea**: data curation (equal), formal analysis (supporting), investigation (equal), methodology (equal), supervision (equal), writing – review & editing (equal). **Irene Rodriguez Fernandez**: formal analysis (supporting), investigation (equal), methodology (equal), resources (equal), writing – review & editing (equal). **Dario Ferreira Sanchez**: formal analysis (supporting), investigation (equal), methodology (equal), resources (equal), validation (equal), writing – review & editing (equal). **Pernilla Eliasson**: conceptualization (supporting), methodology (equal), resources (equal), supervision (supporting), writing – review & editing (equal). **Maria Pierantoni**: conceptualization (equal), data curation (supporting), formal analysis (supporting), supervision (equal), validation (equal), writing – review & editing (equal). **Hanna Isaksson**: conceptualization (equal), funding acquisition (lead), methodology (equal), project administration (lead), resources (equal), supervision (lead), validation (equal), writing – review & editing (equal).

## Funding

This study was supported by H2020 European Research Council (101002516), Schweizerischer Nationalfonds zur Förderung der Wissenschaftlichen Forschung (310030E_188993), European Union's Horizon 2020 through the European Soft Matter Infrastructure (731019).

## Conflicts of Interest

The authors declare no conflicts of interest.

## Supporting information

Supplementary Material

## Data Availability

The data that support the findings of this study are available from the corresponding author upon reasonable request.
