# Supplementary material for: Three‐Dimensional Characterization of the Collagen–Hydroxyapatite Interaction During Heterotopic Ossification in Healing Rat Achilles Tendons
Source: Small Sci. 2026 Jun 20;6(6):e202500644. doi: 10.1002/smsc.202500644 (PMC13285116; doi:10.1002/smsc.202500644)
Supplement: Supplementary file 1 — Supplementary Material [file SMSC-6-e202500644-s001.pdf]

Supplementary material:

Three-dimensional characterization of the collagen-hydroxyapatite interaction during heterotopic ossification in healing rat Achilles tendons

Kunal Sharma[1,*], Isabella Silva Barreto[1], Hector Dejea[1,2], Irene Rodriguez Fernandez[3,4], Dario Ferreira Sanchez[3], Pernilla Eliasson[5,6,7], Maria Pierantoni[1], Hanna Isaksson[1]

[1] Department of Biomedical Engineering, Lund University, Lund, Sweden

[2] MAX IV Laboratory, Lund University, Lund, Sweden

[3] Center for Photon Science, Paul Scherrer Institute, Villigen, Switzerland

[4] Institute for Biomedical Engineering, ETH Zürich, Zürich, Switzerland

[5] Department of Biomedical and Clinical Sciences, Linköping University, Linköping, Sweden

[6] Department of Orthopaedics, Sahlgrenska University Hospital, Gothenburg, Sweden

[7] Department of Orthopaedics, Sahlgrenska Academy, Gothenburg University, Gothenburg, Sweden

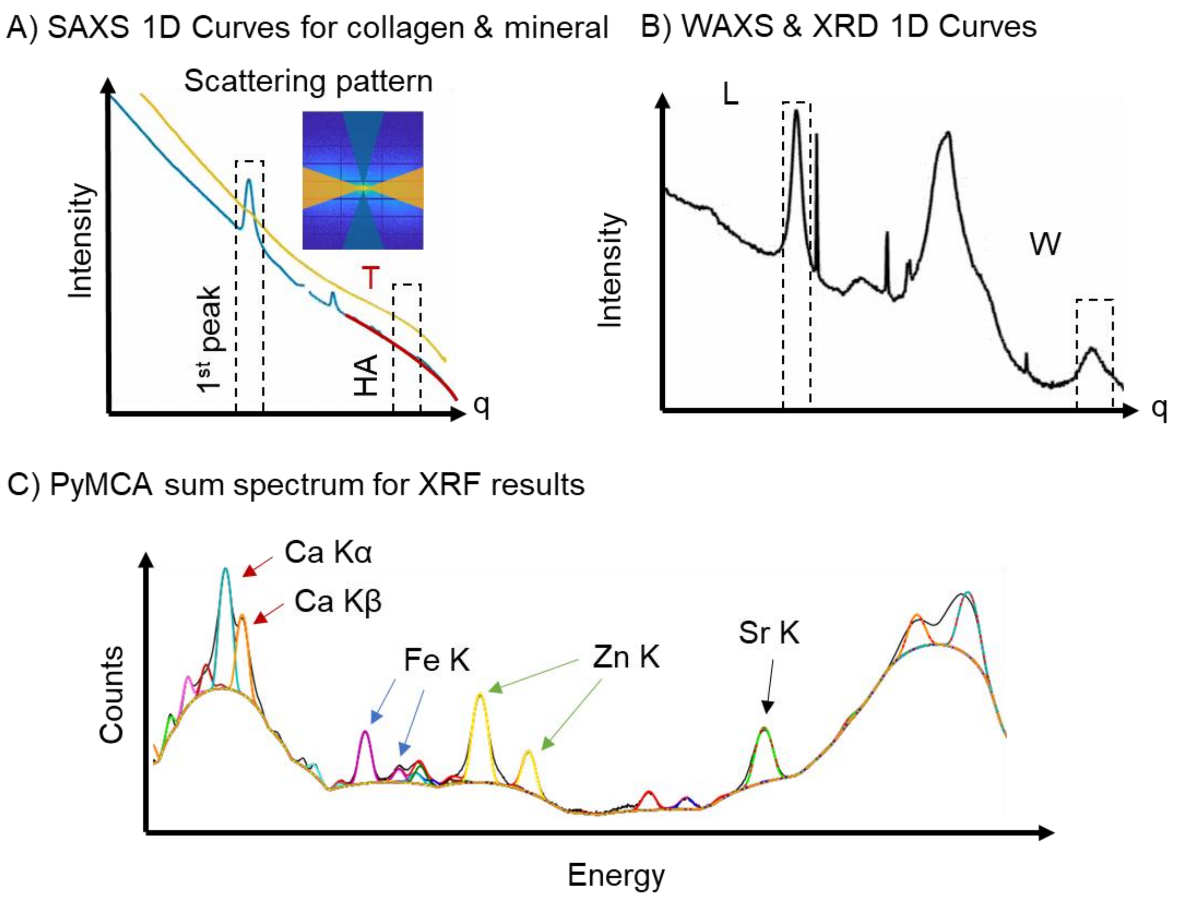

Supplementary Figure S1: X-ray scattering analysis steps. A) small-angle X-ray scattering with collagen information extracted from the 3rd collagen peak (dashed box), and mineral orientation for SASTT (dashed box), and mineral particle thickness based on red line fit (T). B) Wide-angle X-ray scattering and X-ray diffraction 1D curves were analyzed to provide L (crystallite dimension along the c-axis) and W (crystallite dimension along the ab-plane) shown as dashed boxes in the plot. C) PyMCA spectra for the HO deposits with trace element peaks identified.

Supplementary Table S2: Acquisition parameters for SASTT/WASTT for all samples.

	3-week	6-week	12-week	Bone
**Field of view (mm)**	1.9 x 1.5	1.7 x 1.1	1.1 x 0.8	0.7 x 0.6
**Tilt angle α (Δdeg)**	6.43	7.5	7.5	9
**Rot. Angle (Δdeg)**	3.75	4.5	4.5	5.625
**Total scan time (h)**	49.8	28.3	14.9	5.6

Supplementary Table S3: Acquisition parameters for 3D-XRD/XRF for all samples.

	3-week	6-week	12-week	Bone
**Field of view (μm)**	400 x 100	330 x 100	400 x 100	550 x 70
**Rot. Angle (Δdeg)**	1.35	1.64	1.35	1
**Total scan time (h)**	28.3	19.3	28.3	36.9

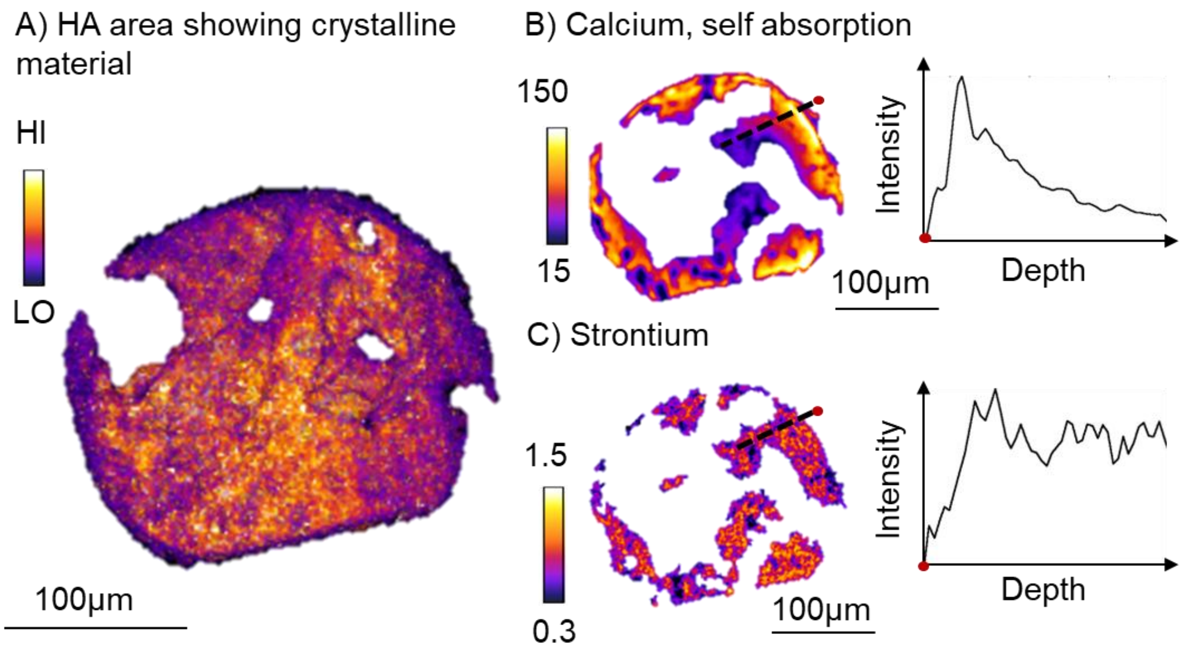

Supplementary Figure S4: Calcium absorption visualized from reconstructed 3D-XRF data. A) HA area in 3D based on 3D-XRD show presence of crystalline material. B) Calcium self-absorption shown on a 2D slice , and a line profile (dashed black line). C) Strontium (used as substitute for Calcium) shown on a 2D slice, and a line profile (dashed black line) of the same region (dashed line).

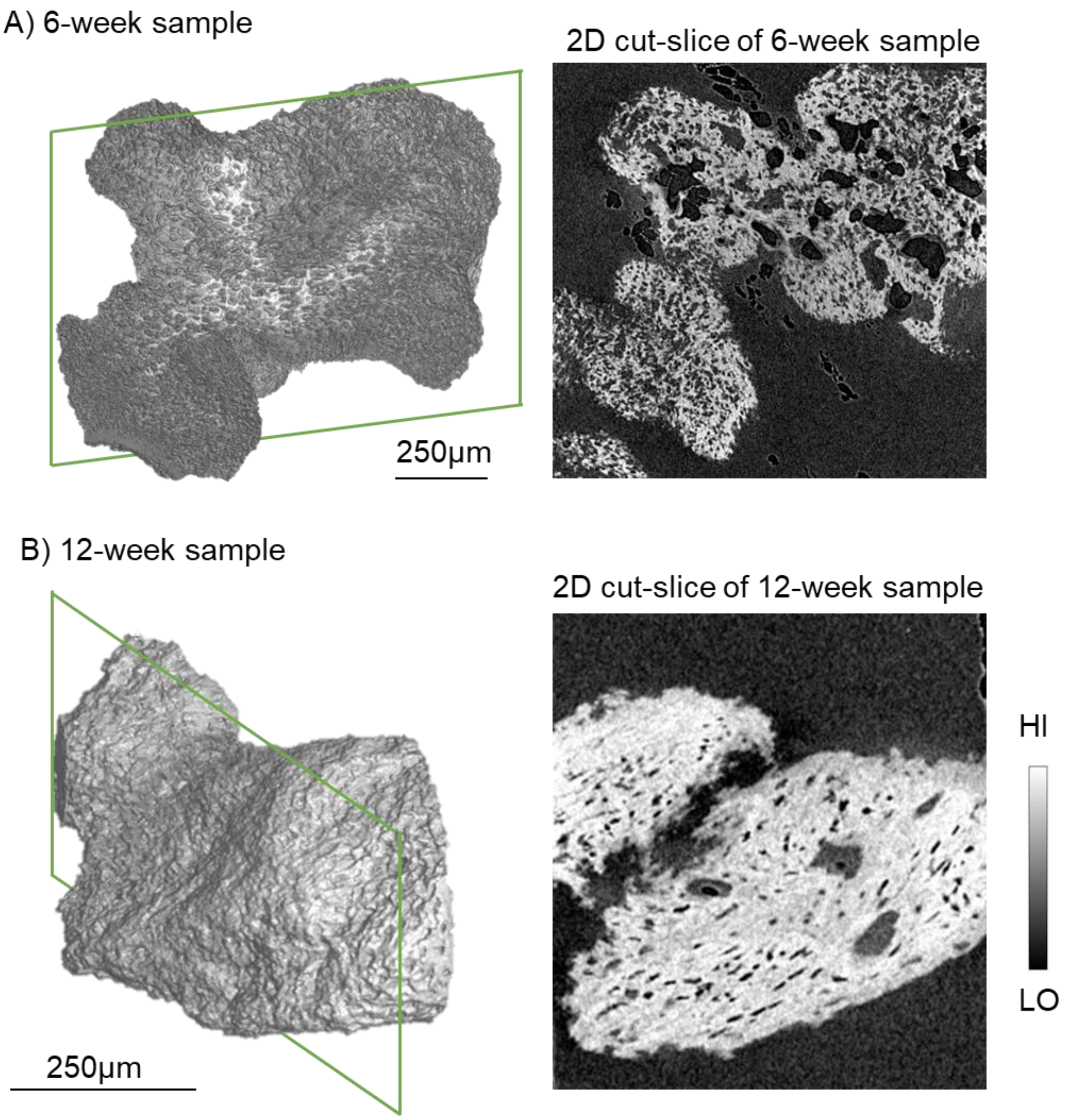

Supplementary Figure S5: Micro-computed tomography of 12-week and 6-week sample, with threshold (LO = 20,000 and HI = 45000, 32bit image). With corresponding 2D slices from the cut plane (green lines).

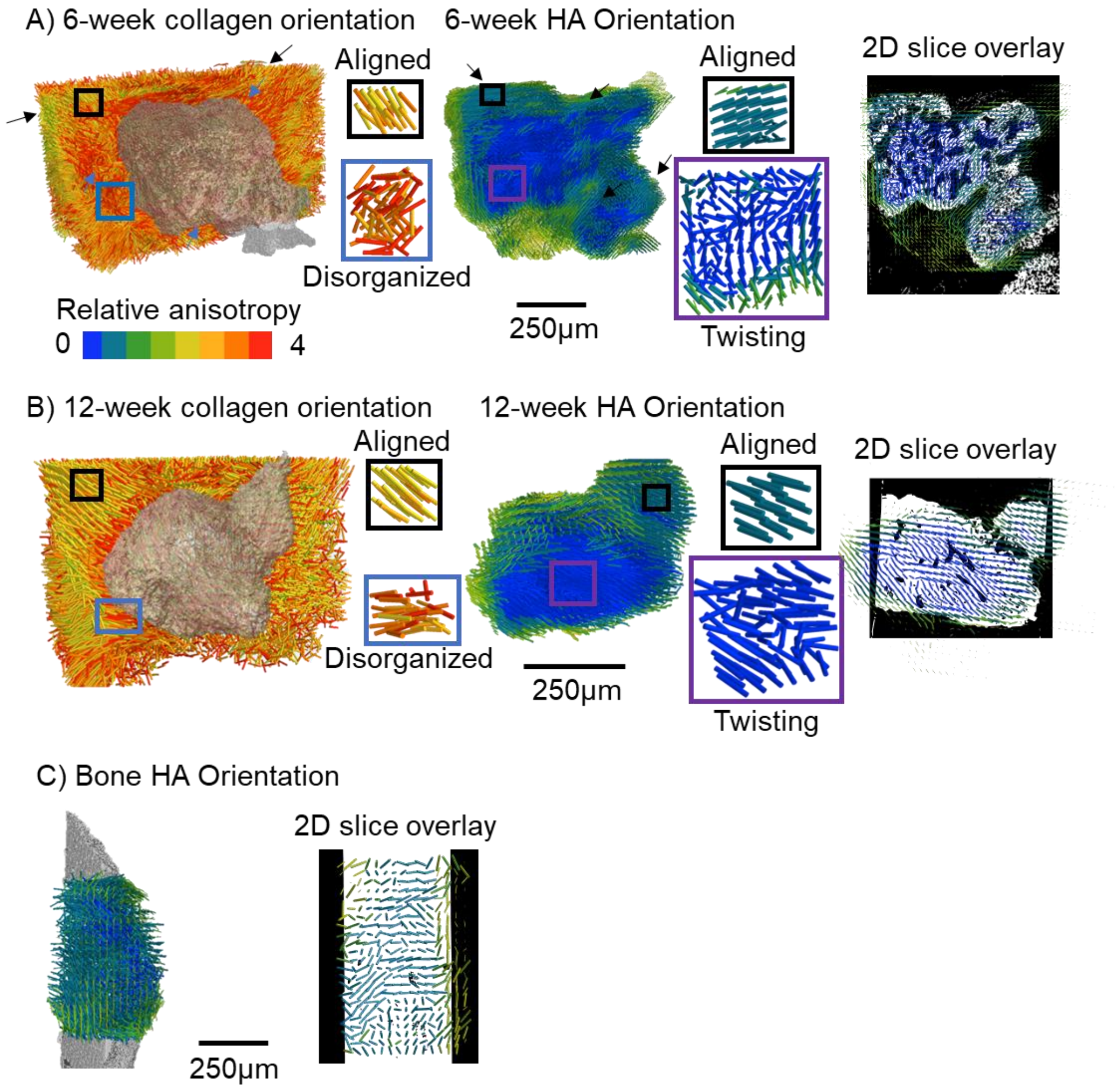

Supplementary Figure S6: Glyph render of SASTT results showing main orientation (glyph direction), mean scattering size (glyph size) relating to amount of material present, and relative anisotropy (color scale) exemplifying the degree of alignment for collagen, and HA for A) 6-week, B) 12-week post rupture samples, and C) bone reference. Collagen orientation is overlayed on their respective microCT volumes, and 2D slice from tomography results is used to demonstrate the orientation changes within the mineralized tissue.

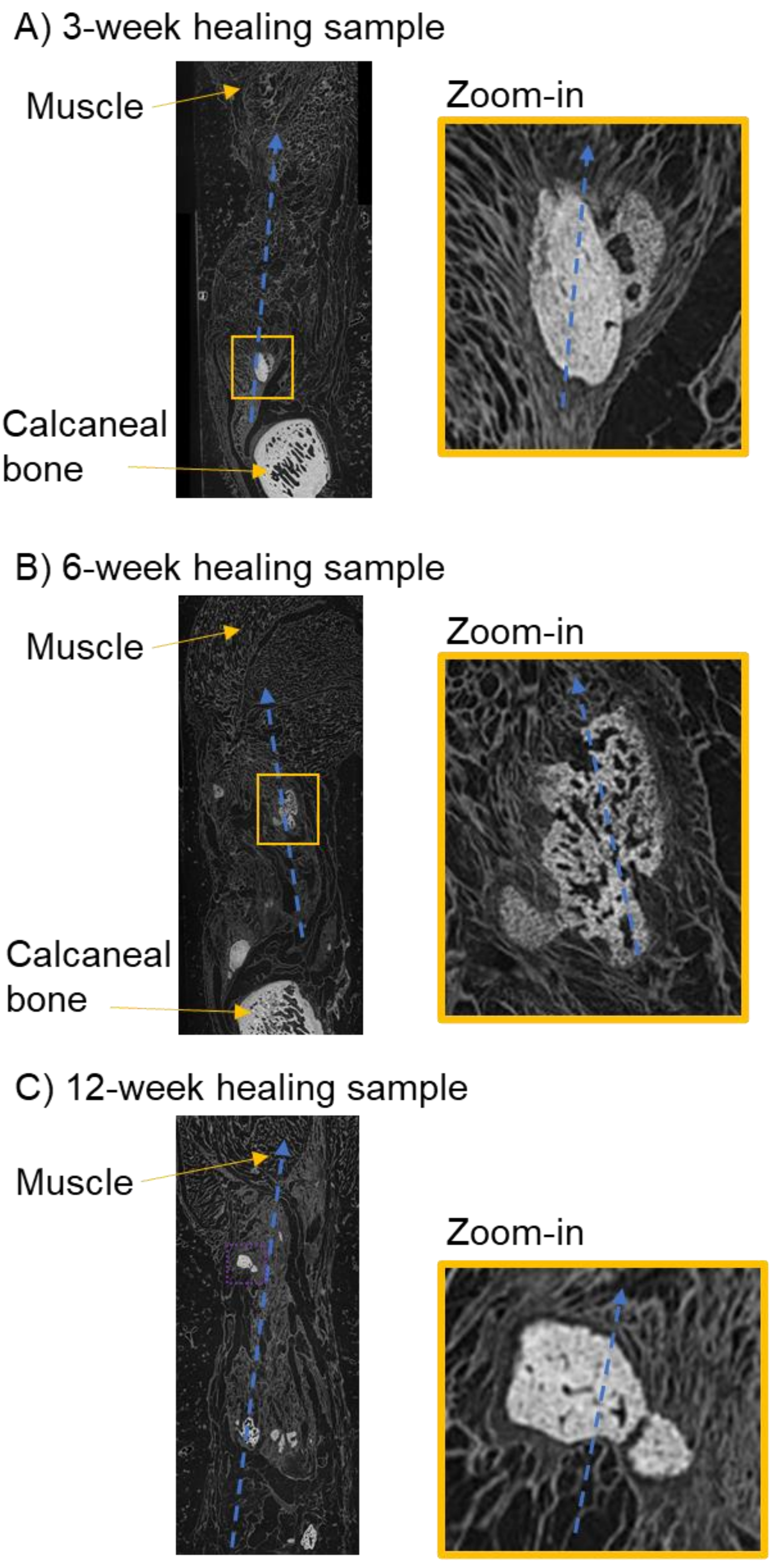

Supplementary Figure S7: Previously acquired tomography images at Diamond Light Source (DLS) that formed the basis for the analysis by Perantoni et al (21). It shows the entire tendon volume of the healing specimens used in the study and the respective HO deposits selected for further analysis. The dashed-line (blue) shows the general tendon direction used as a reference for SASTT analysis.

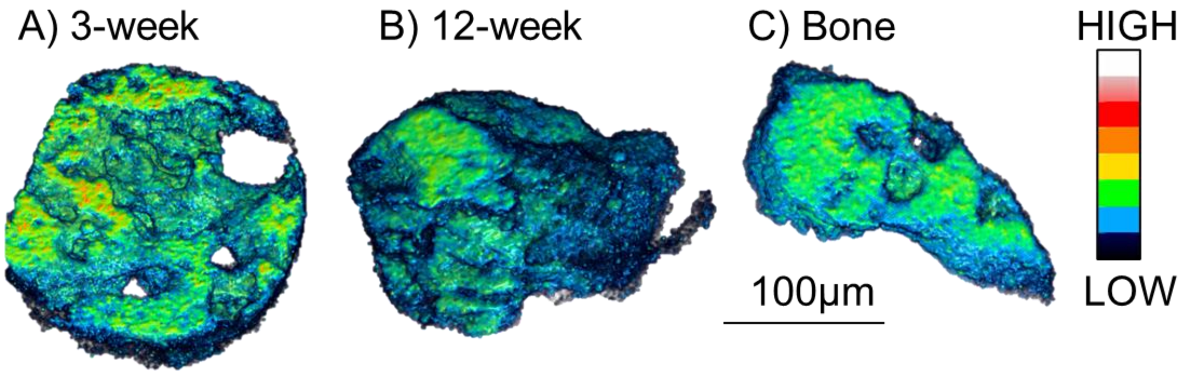

Supplementary Figure S8: Reconstructed HA area based on L x W, with the [002] reflection (L) and [310] reflection (W) from the 3D-XRD acquisition for A) 3-week, B) 12-week samples and C) bone reference.

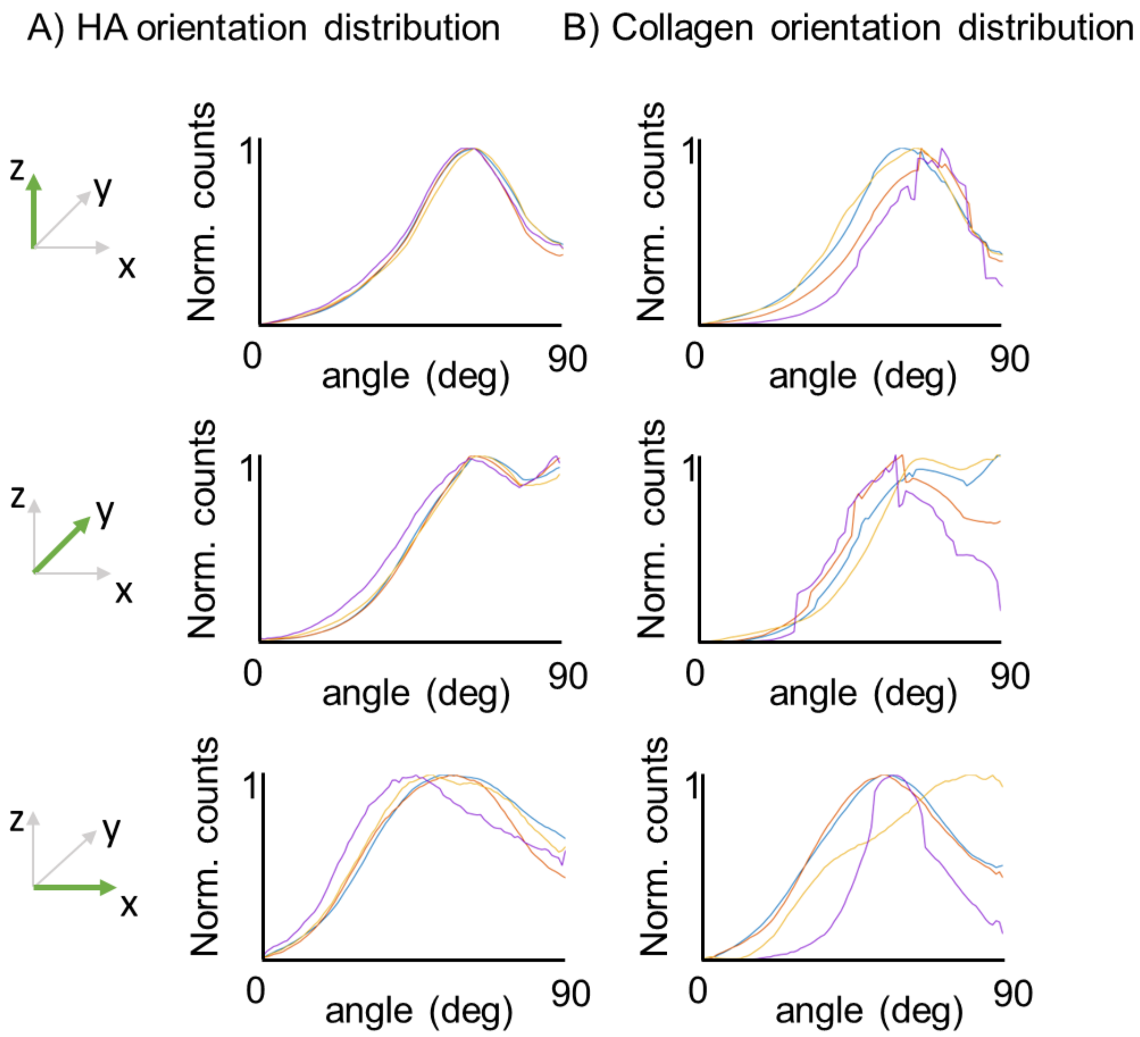

Supplementary Figure S9: Orientation distribution from the SASTT analysis of the A) HA crystal signal, and B) Collagen signal along three different reference directions: x (1,0,0), y (0,1,0), and z (0,0,1).
